# Intraepithelial and Interstitial Deposition of Pathological Prion Protein in Kidneys of Scrapie-Affected Sheep

**DOI:** 10.1371/journal.pone.0000859

**Published:** 2007-09-12

**Authors:** Ciriaco Ligios, Giovanna Maria Cancedda, Ilan Margalith, Cinzia Santucciu, Laura Madau, Caterina Maestrale, Massimo Basagni, Mariangela Saba, Mathias Heikenwalder

**Affiliations:** 1 Istituto Zooprofilattico Sperimentale della Sardegna, Sassari, Italy; 2 Department of Pathology, Institute of Neuropathology, UniversitätsSpital Zürich, Zürich, Switzerland; 3 Prion Diagnostica Srl, Rho, Italy; Roslin Institute, United Kingdom

## Abstract

Prions have been documented in extra-neuronal and extra-lymphatic tissues of humans and various ruminants affected by Transmissible Spongiform Encephalopathy (TSE). The presence of prion infectivity detected in cervid and ovine blood tempted us to reason that kidney, the organ filtrating blood derived proteins, may accumulate disease associated PrP^Sc^. We collected and screened kidneys of experimentally, naturally scrapie-affected and control sheep for renal deposition of PrP^Sc^ from distinct, geographically separated flocks. By performing Western blot, PET blot analysis and immunohistochemistry we found intraepithelial (cortex, medulla and papilla) and occasional interstitial (papilla) deposition of PrP^Sc ^in kidneys of scrapie-affected sheep. Interestingly, glomerula lacked detectable signals indicative of PrP^Sc^. PrP^Sc^ was also detected in kidneys of subclinical sheep, but to significantly lower degree. Depending on the stage of the disease the incidence of PrP^Sc^ in kidney varied from approximately 27% (subclinical) to 73.6% (clinical) in naturally scrapie-affected sheep. Kidneys from flocks without scrapie outbreak were devoid of PrP^Sc^. Here we demonstrate unexpectedly frequent deposition of high levels of PrP^Sc^ in ovine kidneys of various flocks. Renal deposition of PrP^Sc^ is likely to be a pre-requisite enabling prionuria, a possible co-factor of horizontal prion-transmission in sheep.

## Introduction

Scrapie, a fatal, transmissible spongiform encephalopathy (TSE) of sheep and goat caused by prions, has been intensively studied for more than a century [1]. It is broadly accepted that scrapie is caused by prions, as are bovine spongiform encephalopathy (BSE), Creutzfeldt-Jakob disease (CJD) of humans, including those of sporadic, familial, iatrogenic and variant etiology, chronic wasting disease (CWD) of deer, elk, and moose, and a variety of rarer diseases of several other animal species. Prions are thought to mainly consist of PrP^Sc^, a misfolded and aggregated form of the cellular prion protein (PrP^C^) [2] and are used as a surrogate marker for prion affected animals. Many fundamental aspects of peripheral and central scrapie pathogenesis however are yet not understood. A particular important question relates to those factors permitting horizontal transmission of scrapie within sheep flocks. Although the main target of prion-induced pathology is the central nervous system (CNS), PrP^Sc^ can be readily demonstrated in the lymphoreticular system (LRS) in many TSEs [3], [4]–[5], [6]. Moreover, PrP^Sc^ and/or prion infectivity were demonstrated to accumulate at extraneural and extralymphatic sites, including skeletal muscle, mammary gland, placenta and salivary gland in sheep [7], [8]–[9], [10] as well as skeletal muscle [11], [12]–[13], [14] and blood in man [15], [16] and mice [17] or in saliva [10] and skeletal muscles of deer [18]. Yet it is difficult to envisage how tissues that are not exposed to the outer environment might contribute to prion spread among sheep in a flock. Blood was long thought to harbor very little-if any-prion infectivity, yet this assumption was shaken by the efficient blood-borne sheep-to-sheep transmission of scrapie [19], cervid [20] and BSE prions [21] as well as blood-borne transmission incidents of variant CJD (vCJD) between humans [15], [16], [22]. In mice, sheep and deer, chronic lymphocytic inflammation can shift the distribution pattern of PrP^Sc^ or prion infectivity to non-lymphoid organs, being they in the kidney of mouse and deer [23], [24], liver and pancreas of mouse [25], and mammary gland of sheep [8]. Extraneural deposition of PrP^Sc^ appears to become prominent in Creutzfeldt-Jakob disease when PrP^C^ is abundantly available as a substrate, as demonstrated in muscle with inclusion body myositis containing macrophages and lymphocytes [13]. In many instances ectopic prion infectivity and/or PrP^Sc^ was detected in pre-clinically prion-infected individuals [23], [25]. Besides enabling high renal prion loads, chronic lymphocytic inflammation in kidney combined with pathological alterations of the renal filtration apparatus was demonstrated to induce prionuria in both preclinical and terminally scrapie-sick mice [25]. The latter studies yielded the first evidence that excretory organs, when affected by chronic inflammation, can release prion infectivity into the environment. Alternatively, considering that in physiological conditions prions or PrP^Sc^ have been detected in blood of sheep and hamsters affected by scrapie [21], [17], prionuria could occur in the absence of renal immunopathological changes [26]. This last data together with the recent finding of deposition of PrP^Sc^ in the salivary glands of sheep with scrapie [10] accentuate that prionuria may occur, hence contributing in horizontal scrapie transmission. In order to validate this hypothesis, as a first step, we investigated kidneys of experimentally and naturally scrapie-affected sheep derived from distinct scrapie outbreaks.

## Results

### Conventional and Sodium phosphotungstate (NaPTA)-Western blot analysis

Spongiosis, astrogliosis and PrP^Sc^ deposition were found by means of histopathological and immunohistochemical analysis in the brain of all analyzed 72 clinically scrapie-affected Sarda sheep. In addition, presence of PrP^Sc^ was confirmed by conventional and NaPTA-Western blot analysis in the brain, and by immunohistochemistry in the lymphoid tissues (e.g. spleen and palatine tonsils). Identical analysis was performed in sheep lacking clinical signs (sheep from Sardinian, scrapie-free flocks n = 30; sheep from scrapie-affected flocks from Abruzzo and Sardinia n = 67) and did not lead to the detection of PrP^Sc^ in brains and tonsils.

Histopathological examination demonstrated the presence of focal interstitial inflammatory changes, moderate tubular degeneration as well as interstitial fibrosis in some kidneys from the 72 clinically scrapie-affected sheep. However, the damage was moderate in all cases and no overall differences were found when animals belonging to the different groups were compared to the controls (Table 1).

**Table 1:** Number (N), age, PrP genotype and clinical status of the sheep included in the study.

Scrapie-affected sheep						
*Region*	*N flocks*	*N sheep*	*Genotypes (N)*	*Infection*	*Age (years)*	*Clinical signs*
Sardinia	14	72	ARQ/ARQ (63) ARQ/AHQ (9)	Natural	2 to 5	Yes
Sardinia	1	9	ARQ/ARQ	Experimental	2	Yes
Abruzzo	1	3	ARQ/ARQ	Natural	1.5 to 4	Yes
Sardinia	1	11	ARQ/ARQ	Natural	2 to 5	No
**Scrapie negative sheep**						
Sardinia	2	30	ARQ/ARQ (10)	No	2 to 5	No
			ARQ/ARQ (14)			
			ARR/ARR (3)			
			AHQ/AHQ (1)			
			ARQ/ARH (1)			
Sardinia	1	49*	ARQ/ARQ	No	2 to 5	No
Abruzzo	1	18*	ARR/ARR (3)	No	1.5 to 7	No
			ARR/ARQ (8)			
			ARQ/ARQ (6)			
			ARQ/VRQ (1)			

Sheep are grouped as scrapie-affected sheep or negative control. The number (N) of the flocks from which the sheep were selected is also indicated.

* Sheep from scrapie-affected flock

The analysis of PrP^Sc^ deposition in kidneys from the 72 clinically scrapie-affected Sarda sheep indicated 53 (73.6%) or 52 (71.2%) positive cases by using Western blot analysis after NaPTA precipitation or conventional Western blot analysis, respectively. The scrapie sheep positive for PrP^Sc^ in kidney were evenly distributed through all flocks investigated in Sardinia. In contrast, all 79 kidney homogenates derived from control Sarda sheep (49 from scrapie affected sheep and 30 from scrapie-free sheep) were negative for PrP^Sc^ as analyzed by NaPTA precipitation and conventional Western blot analysis. Results resemble at least 2 or 3 independent Western blot analyses after NaPTA precipitation with independent regions of the sheep kidneys.

Three out of the 11 (27%) kidneys from asymptomatic sheep collected from the Sardinian scrapie-affected flock were PrP^Sc^ positive by means of conventional Western blot analysis (Table 1 and 2). Nevertheless, the immuno-histochemical profile and the histopathological features of the obex from these sheep were indicative of clinical stage.

**Table 2:** Number (N) of examined Sarda breed sheep, with or without clinical signs, displaying PrP^Sc^ in the kidney, indicated as percentage (%).

Clinically affected sheep		
*Number of examined sheep*	Number of positive sheep for conventional Western blotting PrP^Sc^ in the kidney (%)*	*Infection*
72	52 (71.2%)	Natural
**Number of clinically healthy sheep**		
11	3 (27%)	Natural

The results are only referred to conventional Western blot examinations.

All the 3 scrapie clinical sheep from Abruzzo were positive for PrP^Sc^ in brain. In addition, NaPTA-Western blot analysis of the renal homogenates of these 3 sheep demonstrated the presence of PrP^Sc^ in 2 of them. Oppositely, renal homogenates from all healthy sheep (n = 18), coming from the same flock, were negative for PrP^Sc^.

These data demonstrate that the incidence of renal PrP^Sc^ deposition in sheep rises with the clinical stage of disease and underline that also subclinical sheep can accumulate PrP^Sc^ in kidney.

Finally, we set out to investigate whether the same phenomenon of renal PrP^Sc^ deposition occurs in experimentally infected scrapie terminal Sarda sheep. Again, in contrast to controls that were negative for PrP^Sc^ in kidney, we detected PrP^Sc^ in 2 out of 9 clinically scrapie sick sheep by conventional Western-blotting and 6 out of 9 by NaPTA-Western blot.

**Figure 1:**
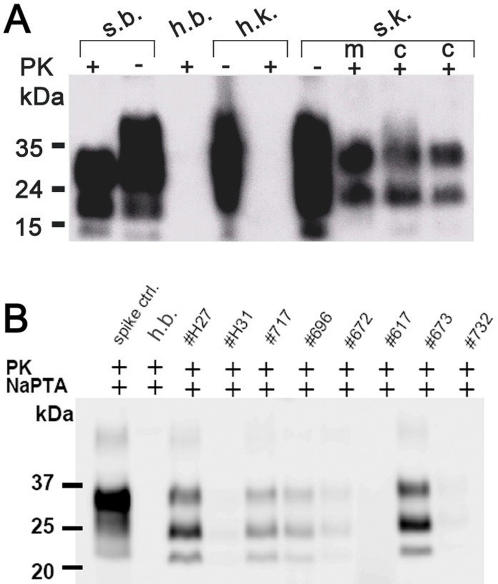
Western Blotting PrP^Sc^ deposition in scrapie-affected Sarda sheep. (A) Detection of PrP^Sc^ by conventional Western blot in the cortical and medullar part of kidneys. Brain and kidney homogenates were treated with (+) or without (−) proteinase K (PK), amounts of kidney and brain represented in each line are 1mg and 1g respectively. S.b. = brain derived from a naturally scrapie-sick sheep; h.b. = brain derived from a scrapie-free sheep control; h.k. = kidney derived from a scrapie-free sheep control, s.k. = kidney derived from a naturally scrapie-sick sheep. kD = kilo Dalton, M = medulla, C = cortex. (B) Detection of PrP^Sc^ by NaPTA Western blot in kidney homogenate derived from different Sarda sheep. Substantial individual variation of the Western blotting signal was observed among the sheep examined. Spike control = brain homogenate derived from a scrapie–sick sheep spiked into negative control kidney homogenate. h.b. = brain derived from a scrapie-free sheep control. 2000–4000 µg of renal homogenate were used as a starting material to perform NaPTA precipitation.

In order to investigate if both cortical and medullary regions (cortex and medulla) of the kidney display PrP^Sc^ we separately performed NaPTA and conventional Western blot analysis in the respective areas of randomly selected kidneys from Sardinia and Abruzzo sheep. Even though we witnessed apparent variation in the amount of cortical and medullary PrP^Sc^, a positive signal was found in both regions of all positive kidneys investigated (Figure 1A). Variations in the intensity of the kidney-derived PrP^Sc^ signal were observed. We hypothesized that the Western-blotting signals become more intense in kidneys from sheep in relative later stage of scrapie. Further, we screened all renal homogenates by NaPTA-Western blot analysis (Figure 1B).

### Paraffin-embedded tissue (PET) blot analysis and Immunohistochemical analysis

**Figure 2:**
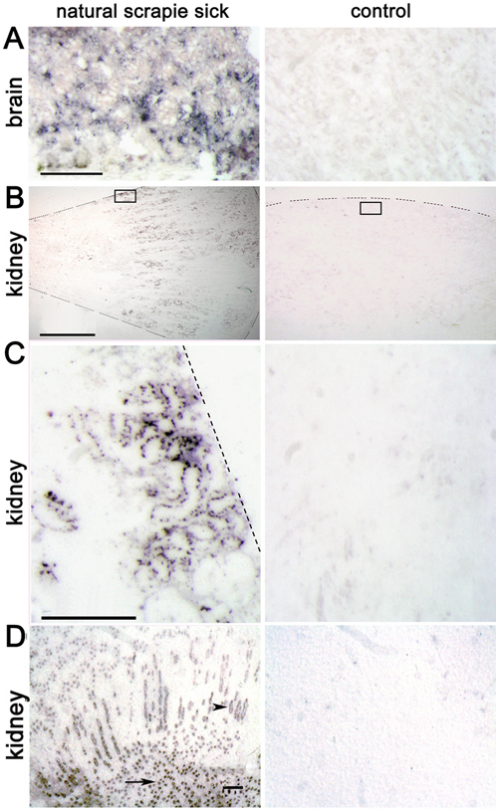
PET blot analysis identifying PrP^Sc^ in the tubular structures of the cortex and medulla in naturally scrapie-sick Sarda sheep. (A) Brains of scrapie-sick (left) and healthy sheep (right) as a control for PET blot analysis procedure. Scale bar = 100 µm. (B) Low magnification of transverse paraffin sections from kidneys of a naturally scrapie-sick and a healthy sheep demonstrating PrP^Sc^ in the medullary and cortical regions of the kidney. Dotted lines indicate the locus of the kidney capsule or the edge of the tissue section. The small transparent box indicates the region shown at higher magnification and resolution in figure C. Scale bar = 1 cm. (C) PrP^Sc^ deposits and tubular structure fit together in the cortical part of the kidney in scrapie-sick sheep. Scale bar = 100 µm. (D) PrP^Sc^ is also found to a high degree in the collecting ducts of the medulla (arrowhead) as well as in the papillae (arrow) of kidneys derived from naturally scrapie infected sheep. Scale bar = 30 µm.

In order to better define the localization and to verify the presence of PrP^Sc^ in kidneys with different methods, we performed PET blot analysis on paraffin sections of natural scrapie-sick and control sheep. As indicated PrP^Sc^ is clearly detectable in the tubular structures of the renal cortex and medulla (Figure 2B and C), including the papillae (Figure 2D). In the medulla the rate of immunostained tubular structures (collecting ducts) was higher than in the cortex, where immuno-positive signals were restricted to a few convoluted tubules. All negative animals were devoid of PK resistant PrP^Sc^ in the kidney.

**Figure 3:**
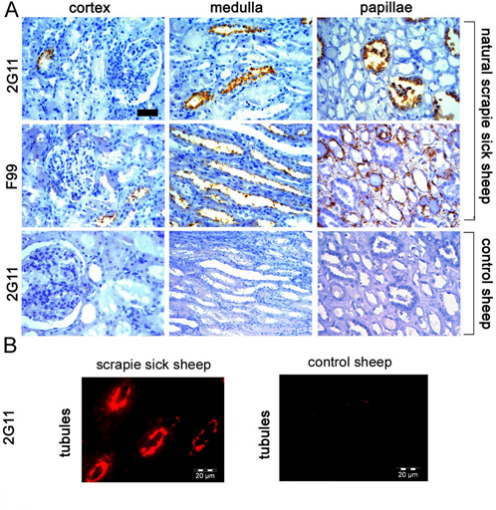
PrP^Sc^ detected by immunohistochemistry in scrapie-affected Sarda sheep. (A) Immunohistochemical analysis of the cortex, medulla and papillae of naturally scrapie-sick sheep and control sheep derived from scrapie-free flocks. Antibodies 2G11 and F99 were used. Scale bar = 30 µm. (B) Immunofluorescence using 2G11 identifies tubular deposits in the medullar-cortical junction of the kidney of a scrapie-sick sheep. Scrapie-free control is devoid of detectable positive signal.

Immunohistochemical and immunofluorescence analysis of consecutive paraffin sections from randomly selected kidneys of Sardinian naturally scrapie affected sheep (n = 33) demonstrated that 23 out of the 33 kidneys showed deposits of PrP^Sc^ positive for F99 antibody or for 2G11 (Figure 3A and B). Immunohistochemistry results were not always congruent with results gained from NaPTA and conventional Western blot analysis, though the rate of PrP^Sc^ positivity obtained by immunohistochemistry was comparable to Western blot analysis (69.6% *versus* 73.6% and 71.2%).

Moreover, all 23 kidneys accumulated PrP^Sc^ in both the cortex and the medulla, including the collecting ducts and the papillae, as demonstrated by PET blot and immunohistochemistry analysis (Figures 2 and 3). Kidney paraffin sections from control sheep remained negative for staining by 2G11 and F99 (Figures 2 and 3). The intensity of PrP^Sc^ staining varied among the different kidneys and regions (cortex, medulla, papillae). Notably, the immunohistochemical patterns were different depending on the antibody used. Our analysis demonstrated granular PrP^Sc^ deposits with the 2G11 antibody exclusively within the epithelial tubular cells of the convoluted tubules and the collecting ducts (Figure 3A). On the contrary, immunostaining with F99 showed presence of PrP^Sc^ staining on the surface of tubular cells of convoluted tubules (cortex) and in the collecting ducts (medulla and papillae) (Figure 3A). Moreover, with the same antibody in two cases PrP^Sc^ staining was demonstrated in the intertubular spaces of the renal papillae displaying a granular deposition, mostly around the basal membrane of the tubular structures (Figure 3A). Surprisingly, in these two cases, serial renal sections of the same area identified PrP^Sc^ granular deposits only within the tubular cells of the collecting ducts when probed with the 2G11 antibody. With both antibodies in the medulla the rate of immunostained tubular structures was higher than in the cortex, where immuno-positive signals were restricted to a few convoluted tubules.

### Quantification of renal PrP^C^ and PrP^Sc^ in scrapie-sick and control sheep

**Figure 4:**
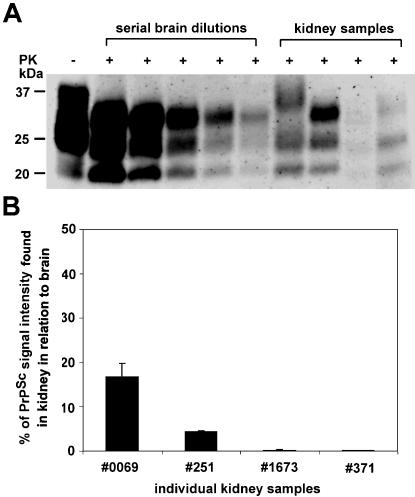
Quantification of PrP^Sc^ in renal homogenates of naturally scrapie-sick sheep. (A) Conventional Western blot analysis of various brain dilutions after proteinase K (PK) digestion. Undigested brain is loaded to control for PK digest (lane 1). Kidney homogenates from independent scrapie sick animals were loaded (lanes 7–10). (B) Relative quantification of PrP^Sc^ signal intensity found in kidney homogenates in relation to brain derived PrP^Sc^. Our results indicate that the highest amounts of renal PrP^Sc^ detected were appr. up to 20% of the PrP^Sc^ signal detected in brain.

Furthermore we determined the relative amount of renal PrP^Sc^ in scrapie-sick sheep compared to brain derived PrP^Sc^. Serial dilution experiments of PK digested brain homogenates spiked into kidneys from healthy sheep were performed, thereby generating a standard curve for the amount of PrP^Sc^/µg of brain tissue from various brain samples (Figure 4). These results were compared to a selected pool from independent animals of kidney homogenates loaded on the identical blot. After densiometric analysis, the relative amount of PrP^Sc^ found in µgs of kidney homogenate was quantified and blotted as percentage of PrP^Sc^ positive signal intensity found in µgs of brain homogenates (Figure 4). The relative amount of PrP^Sc^ found in kidney ranged from 5–10 fold to 100–500 fold lower than in brain. Even though 100–500 fold less PrP^Sc^ was detected in kidney when compared to brain, PrP^Sc^ was still detectable by NaPTA and conventional Western blot analysis.

### Genetic analysis

Sequencing of the complete PrP gene (*PRNP*) of clinically scrapie-affected 16 sheep (8 of which harbouring PrP^Sc^ in the kidney) confirmed the ARQ/ARQ genotype for all of them and did not demonstrate the existence of additional polymorphisms. All remaining genotypes of sheep were determined at codon 136, 154 and 171 (Table 1).

## Discussion

Our study involved a high number of animals, providing good evidence that PrP^Sc^ can be found to high degree in the medullary and the cortical regions of kidneys derived from ARQ/ARQ scrapie-sick sheep. Moreover, we can demonstrate that the incidence of renal PrP^Sc^ deposition increases with the stage of disease.

Presence of PrP^Sc^ in kidney was detected in renal glomerula of cats [27] and cheetah [28] affected by feline spongiform encepaholpathy by the means of immunohiostochemical analysis. However, both findings are observations without Western blot or Histoblot and PET blot analysis, which are essential to prove the effective presence of PrP^Sc^, a surrogate marker of prion infectivity. In addition, Herzog and colleagues [29] described PrP^Sc^ accumulation in kidney of primates experimentally inoculated with BSE by using an ELISA-based assay. However, also this technique did not allow to localize PrP^Sc^ nor to characterize renal PrP^Sc^ biochemically. In contrast to the described PrP^Sc^ deposition pattern in cats or cheetah, we do not find PrP^Sc^ positive signals in glomerula. This stresses the differences between various species in renal PrP^Sc^ deposition and may underline possible consequences for enabling horizontal prion transmission in particular species. By solely using immunohistochemistry Sisó et al., [30] showed the presence of pathological prion protein, which they called PrP^d^. The authors found PrP^d^ only in the renal medulla of scrapie sick sheep. By conventional and NaPTA-Western blot analysis we were able to detect PrP^Sc^ in cortex and medulla in similar amounts, whereas immunohistochemistry revealed stronger signals in medulla than in the cortex. In this study, conventional and NaPTA-Western blot analysis detected a comparable prevalence of kidneys positive for PrP^Sc^. The observation of cortical PrP^Sc^ deposition as well as the higher incidence of PrP^Sc^ found in Sardinian and Abruzzo flocks when compared to the publication by Sisó et al. [30], might result from differences in the flock origin, different scrapie strain or scrapie disease progression. In our study, the sequencing of the PrP gene showed that sheep had the same ARQ haplotype, indicating that the renal PrP^Sc^ deposition is not due to polymorphic variations of the PrP gene. Since Sisó et al [30] found a higher presence of PrP^Sc^ in sheep experimentally inoculated with an ARQ/ARQ Suffolk isolate, the deposition of PrP^Sc^ might be mostly influenced by the source of the etiological agent. Further studies are needed to establish whether renal PrP^Sc^ deposition occurs in a similar manner in VRQ/VRQ sheep naturally affected by scrapie.

Most importantly, our survey demonstrated that renal PrP^Sc^ deposition is mostly relegate to the clinical stage of the disease, indeed only 3 out of the 11 asymptomatic sheep harbored PrP^Sc^ in the kidney. In addition the immunohistochemical patterns that were observed in brain of these 3 sheep were indicative of clinical scrapie, suggesting a later stage of the disease.

In contrast to the previously published studies, we conclusively demonstrate for the first time, by using immunohistochemical, immunofluorescent and PET blot analysis, intraepithelial, but not glomerular, deposition of PrP^Sc^ in kidney. However, the deposition patterns detected by immunohistochemistry vary with the epitope specificity of the detecting antibody, indicative of different PrP^Sc^ isoforms or differential processing in the kidney of naturally scrapie-sick sheep. This finding is again in contrast to the previously published study that indicated differences in staining intensity but not in staining pattern [30]. In this case it is reasonable to suppose that differences in immunohistochemical procedures (e.g. formalin fixation, antibodies, etc.) can play an important role.

Indeed, using the same antibodies (F99 and G11) Vascellari et al. [10] found analogous PrP^Sc^ deposition patterns in the salivary glands of ARQ/ARQ sheep with scrapie. This might suggest, on one side, a similar mechanisms of PrP^Sc^ accumulation in the kidney and in the salivary glands, on the other, a similar role of the filtrating epithelial cells and of the secreting epithelial cells in spreading prions.

Since prion infectivity was demonstrated in blood of sheep [21], in the light of these results it could be hypothesized that blood contains prions which could be filtered and accumulated by the kidney, resulting in renal PrP^Sc^ accumulation. A multitude of experimental paradigms shows that prion diseases can be transmitted from one individual to another. Many of the parameters controlling transmissibility are relatively well understood. Firstly the size of the infectious inoculum, secondly the route, with intracerebral inoculation being more efficient than oral, intraperitoneal and intravenous injection, thirdly the polymorphisms in the *Prnp* gene of both donor and recipient.

For a living organism to initiate horizontal transmission under field conditions of any infectious agent, including prions, the agent must be released from the organism into the environment, through respiratory aerosols, bodily secretions, or excrements. The unsuccessful attempts at eradicating scrapie from Icelandic flocks in the 20^th^ century bear witness to the likelihood of environmental prion contamination. Additional pathways of horizontal transmission of sheep scrapie may include parasitic vectors [31], [32] and placental cannibalism within flocks [9]. The importance of the two latter routes in naturally occurring transmission of field cases, however, is supported by limited evidence.

Previous studies in sheep, humans, and genetically modified mice have pointed to inflammatory conditions as a cofactor enabling prion replication in disparate tissues, including liver, pancreas, skeletal muscle, mammary gland, and kidney [11], [25], [8]. The function of the latter organs is excretion, and indeed chronic lymphofollicular nephritis leads to prionuria in prion-infected mice [24]. As a first step towards investigating the relevance of prionuria in horizontal transmission of scrapie, we undertook to investigate the presence of PrP^Sc^ in sheep kidney.

Work in progress will define the spatiotemporal mechanisms of renal PrP^Sc^ deposition in subclinical and naturally scrapie-sick sheep as well as the epidemiological importance of this finding. Due to prion infectivity detected in sheep blood, it is not surprising that renal prion deposition can occur independent of lymphocytic inflammatory disorders. Potentially, renal prion deposition could be a result of blood borne prions. Prions were recently demonstrated in blood of 50–60% of subclinical and in >80% of terminal hamsters [17]. This could explain why also subclinical sheep could accumulate prions in kidney but to less degree and incidence. Alternatively, ovine kidneys could potentially contain renal cells that are capable of prion replication itself.

It will be extremely important to evaluate whether inflammation can further increase renal deposition of PrP^Sc^ and whether prion infectivity can be detected in kidney and urine of naturally scrapie-sick sheep, during the clinical and preclinical stage. Our data suggest that prionuria might indeed be possible in an unexpected high percentage of affected animals, presenting an important co-factor for horizontal prion transmission.

## Materials and Methods

### Sampling

Our study was carried on experimentally and naturally infected sheep as well as appropriate control sheep located in Sardinia and Abruzzo (Italy). In Sardinia, we set out to investigate naturally scrapie-sick Sarda sheep (n = 72) from 14 distinct flocks as well as experimentally scrapie-sick Sarda sheep (n = 9), inoculated with 25 ml of a 10% brain homogenate (pooled from natural ARQ/ARQ sheep scrapie cases) at 40 days of age. Additionally, to verify whether our hypothesis was valid in different and geographically separated breed we investigated an additional scrapie outbreak in Abruzzo, where we analyzed Comisana-crossed breed sheep with natural clinical scrapie (n = 3), (Table 1).

All the sheep (n = 75) coming from these different sources were sacrificed at clinical stage of the disease. Lastly, to define whether PrP^Sc^ deposition already occurs at asymptomatic stage of the disease, we analyzed 11 asymptomatic scrapie-affected sheep from a single flock located in Sardinia. In addition, 30 control Sarda breed sheep from flocks without scrapie cases and 67 healthy sheep from 2 scrapie affected flocks, located respectively in Abruzzo (n = 18 sheep) and in Sardinia (n = 49 sheep) were analyzed, other details are reported in Table 1.

All the 9 ARQ/ARQ orally infected sheep, after an average incubation period of 650 days (+/−25 days) showed clinical signs of scrapie and were sacrificed at terminal stage (mean clinical course = 27 days). The 75 naturally scrapie-affected sheep with clinical signs were monitored in the open field on a constant basis and sacrificed at terminal stage, approximately 40 days after the onset of the first clinical signs.

From all sheep we collected brain, palatine tonsil, kidney and spleen, which were partly frozen at −20°C or −80°C and partly fixed in 10% neutral buffered formalin. For investigating the regional distribution of PrP^Sc^ in kidneys we separated cortex and medulla by macro-dissecting transversal sections of whole kidneys and used pooled (cortex and medulla) or separated regions for Western blot analysis.

### Conventional WB analysis

Frozen kidney, nervous and LRS tissue samples were thawed and then submitted to an appropriate Western-blotting (WB) protocol for PrP^Sc^ detection. For the examination of kidney, this was carried out by means of a modified Prionics check protocol (Prionics AG, Switzerland), with 1 g of kidney being first incubated in TBS buffer (10 mM Tris HCl, 133 mM NaCl, pH 7.4) containing 1,5 mM CaCl_2_ and 2.5 mg/ml (final concentration) of type XI collagenase 1.6 U/mg (Sigma, USA) for 2 h at 37°C. Samples were subsequently centrifuged at 30 000 g for 2–30 min at 4°C, with the pellets resuspended in the homogenization buffer (20% w/v) which is included in the Prionics check kit (Prionics AG, Switzerland), and homogenized using an automatic system (Fasth PCPM, Italy). A 5000 μl volume of this homogenate was incubated with 500 µl of 10× digestion buffer and proteinase K (50 µg/ml), both of which were also included in the Prionics check kit, at 50°C for 40 min, and the reaction was blocked with 50 µl of digestion stop buffer. After digestion, further concentration steps were performed by centrifugation at 30,000 g for 30 min at 4°C. Pellets were resuspended in 20 µl of 1× NuPage SDS reducing buffer and 10 µl of each solution was loaded on (12%) NuPAGE Novex Bis-Tris Gels 12 wells (Invitrogen, USA) for electrophoresis under constant application of 120 V for 45 min. Electroblotting was performed onto polyvinylidene fluoride membranes (150 V for 1 h). For PrP^Sc^ detection, membranes were incubated over night at 4°C with P4 monoclonal antibody (MoAb) (1∶15,000), which is specific for the aa89-104 of the ovine PrP [33]. After washing with a solution of 10 mM Tris HCl, 133 mM NaCl, and 0.2% Tween 20 (TBST), the secondary Ab diluted 1∶5000 was added to the reaction, with membranes being subsequently washed 4 times for 5 min, equilibrated in luminescence buffer, placed in chemiluminescent substrate (Roche, Switzerland), and finally exposed to an X-Ray film. Examination of the obex was carried out using the Prionics check kit, in accordance with the manufacturer's instructions.

### Western blot analysis with Sodium phosphotungstate (NaPTA) precipitation

10% homogenates of brain and kidney were prepared in 2% Sarcosyl or PBS. Gross cellular debris were removed by centrifugation at 500 g for 1 min. The resulting supernatant was adjusted to 500 µl with PBS, and mixed 1∶1 with 4% Sarkosyl in PBS. Samples were incubated for 15 min at 37°C under constant agitation. Benzonase and MgCl_2_ were added to a final concentration of 50 U/ml and 12.75 mM respectively, and incubated for 30 min at 37°C under continuous agitation. Pre-warmed PTA stock solution (pH 7.4) was added to a final concentration of 0.3% and the sample was incubated at 37°C for 30 min with constant agitation, followed by centrifugation at 37°C for 30 min at maximum speed (14800 g) in an Eppendorf microcentrifuge. The pellet was resuspended in 30 µl 0.1% Sarkosyl in PBS and digested with 30 or 50 µg/ml proteinase K (PK) for 30 min at 37°C with agitation. The sample was heated at 95°C for 5 min in SDS-containing loading buffer before loading onto 10,12 or 16% Novex SDS polyacrylamide gels (Invitrogen, USA), then wet-blotting were performed as described in the previous paragraph using as primary antibody POM1/POM19 or POM19 alone, which bind on distinct epitopes on the globular domain of PrP [34].

### Quantification of total brain or kidney protein

From 10% homogenates of kidney and brain was performed with a BCA™ Protein Assay Kit, according to the manufacturer̀s protocol (23-225 BCA™ Protein Assay Kit, Pierce).

### Quantification of PrP^Sc^ in brain and kidney

To analyze and to quantify PrP^Sc^ in ovine kidney homogenates of natural scrapie sick sheep 250–400 µg total protein of selected kidney samples were digested with proteinase K (50 µg/ml) for 30 min at 37°C and loaded on a pre-casted 12%-SDS gel (NuPAGE). Proteins were transferred to nitrocellulose (Schleicher-Schuell, Germany) by wet-blotting. Membranes were blocked with TBST containing 5% Top Block or 5% bovine serum albumine (BSA) (Juro, Switzerland). As primary MoAb was applied P4 and also POM1 and/or POM19. On the same blot serial dilutions of brain homogenate (2–0.125 µg), spiked into 300 µg kidney protein derived from a sheep without clinical signs, were PK digested, loaded and developed. To control PK digest, undigested and digested brain homogenate as well as a dual color marker were loaded. Blots were developed until the three characteristic, shifted, PK resistant bands appeared in the kidney homogenates. Signals received from serial brain dilutions were quantified in the non-saturated, linear range after development using the VersaDoc imaging system (Biorad) and Quantity one® software (Biorad). Thereby a standard curve was generated, allowing relative quantification of PrP^Sc^/µg of brain or kidney tissue.

### Immunohistochemical techniques

Tissues were formalin-fixed, treated for 1 hr in 98% formic acid, and embedded in paraffin using conventional protocol. For histopathology, nervous and renal specimens were cut into 5 µm-thick sections and stained by haematoxylin and eosin (HE) and then examined by light microscope examination. For immunofluorescence, tissue sections, mounted onto positively charged glass slides, were autoclaved in a citrate buffer (pH 6.1) at 121°C for 10 min to expose epitopes. Slides were immersed in a solution of 0.3% H_2_O_2_ for 10 min at room temperature (RT) to quench endogenous peroxidase activity, rinsed with PBS and nonspecific sites were blocked in 5% BSA in PBS. Slides were then incubated with anti-PrP monoclonal antibody 2G11 followed by an incubation with an Alexa Fluor conjugate 594 secondary antibody. Negative tissue controls included kidneys from ARR/ARR scrapie-negative sheep. For immunohistochemistry, tissue sections were first dried overnight at 37°C, rehydrated and then autoclaved at 121°C for 10 min in a solution of 0.01 M citric acid (pH 6.1). Further steps included utilization of a biotin-streptavidin detection method (Vector Laboratories, Inc., U.S.A.), using the following primary monoclonal antibodies (MoAbs): 2G11 (1 µg/µl) (1: 500) and (1 µg/µl) F99 (1∶800). 2G11 and F99 antibodies have epitopes on aa151-159 and aa220-225 of the ovine PrP, respectively. Immune reactions were visualized by 3-3′-diaminobenzidine (DAB) chromogen solution (Dako, Denmark). Tissue sections from ARR/ARR scrapie-negative sheep were included in each run as control. Another series of controls was set up by omitting the primary MoAbs.

### Paraffin-embedded tissue (PET) blot analysis

2 µm thick paraffin sections were collected on a nitrocellulose-membrane and dried over night at 37°C and just before use for 1h at 56°C. Deparaffinization in Xylol, twice for 5 min, followed by 100% isopropanol, twice for 2 min, 95% isopropanol, once for 2 min and 70% isopropanol (once for 2 min). The membranes were then washed in 0.1% Tween-20 in distilled water for 10 min and air-dried over night. This procedure was followed by a washing step in 1× Tris-buffered Saline with 0.5% Tween-20 (TBST) for 1 hr and a PK digestion (Roche) (250 µg Proteinase K/ml) in digestion buffer (100 mM NaCl, 0.1% Brij 35 in 10mM Tris-HCl, pH 7.8) at 55°C for 8 hrs. PK digestion was followed by washing steps in TBST (3×10 min). Denaturation 3M Guanidinium thiocyatnate (Sigma) in 10 mM Tris-HCl, pH 7.8) for 10 min was followed by further washing steps in 1× TBST (3×10 min) and blocking with 5% low fat milk in 1× TBST (blocking buffer), for 1 hr. Thereafter the primary MoAb POM1 or 2G11 (POM1: 200 ng/ml; 2G11: 500 ng/ml) in 1% low fat milk in 1× TBST was incubated over night at RT. Washing was performed thereafter in 1× TBST (3×10 min) followed by blocking in 1% low fat milk 1× TBST for 5 min. Incubation of the secondary secondary AB (Vector or DAKO D0486, AP-conjugated goat anti mouse; 1:1000) was performed with the membrane for 2 hrs–3 hrs at RT., followed by washing with 5% low fat milk in 1 × (3×10 min). For development of signal Vecstain ABC–AmP (Vector) was incubated for 10 min at RT followed by incubation with BCP/NBT for 30 min at RT. After a final washing step in 5% low fat milk in 1× TBST the blots were air dried and flattened with a glass plate for visual inspection. Alternatively, for development of signal B3 DIG was incubated for 10 min followed by incubation with NBT and BCIP for 1 hr. After a final washing step in distilled water for at least 30 min the blots were air-dried and flattened with a glass plate for visual inspection. As positive and negative controls the brain of terminal scrapie-sick sheep and ARR/ARR sheep were used, respectively.

### Genetic analysis

The PrP genotypes of all investigated sheep were determined focusing on the PrP gene (*PRNP*) polymorphisms at codons 136, 154, and 171, keeping in mind that the polymorphic site which is known to influence susceptibility/resistance to scrapie in Sarda breed sheep is represented by codon 171, with 171 Q/Q homozygous sheep being the most susceptible to disease. DNA was isolated from EDTA-treated blood and submitted to appropriate TaqMan allelic discrimination using ABI Prism (Applied Biosystem, USA), as described elsewhere [35]. Additionally, from 16 scrapie affected sheep, of which only 8 with PrP^Sc^ in the kidney, genomic DNA was isolated from the brain tissue by the DNA isolation kit (Qiagen, USA). PCR amplification of the PrP gene was performed using the following primers: PrP1(+), 5′-ATGGTGAAAAGCCACATAGGCAGT-3′, and PrP2(−), 5′-CTATCCTACTATGAGAAAAATGAG-3′. The PrP1(+) and PrP2(−) anneal at the extreme 5′ and 3′ regions of the PrP-coding sequence, respectively. Amplification reactions were performed for 30 cycles of 30 sec at 94°C, 30 sec at 59°C, and 45 sec at 72°C. PrP gene polymorphisms were detected by DNA sequencing on both strands of the PCR products (Applied Biosystems, USA).
